# Inverse reinforcement learning for intelligent mechanical ventilation and sedative dosing in intensive care units

**DOI:** 10.1186/s12911-019-0763-6

**Published:** 2019-04-09

**Authors:** Chao Yu, Jiming Liu, Hongyi Zhao

**Affiliations:** 1 School of Computer Science and Technology, Dalian University of Technology, Dalian, China; 2 Department of Computer Science, Hong Kong Baptist University, Hong Kong, China

**Keywords:** Reinforcement learning, Inverse learning, Mechanical ventilation, Sedative dosing, Intensive care units

## Abstract

**Background:**

Reinforcement learning (RL) provides a promising technique to solve complex sequential decision making problems in health care domains. To ensure such applications, an explicit reward function encoding domain knowledge should be specified beforehand to indicate the goal of tasks. However, there is usually no explicit information regarding the reward function in medical records. It is then necessary to consider an approach whereby the reward function can be learned from a set of presumably optimal treatment trajectories using retrospective real medical data. This paper applies inverse RL in inferring the reward functions that clinicians have in mind during their decisions on weaning of mechanical ventilation and sedative dosing in Intensive Care Units (ICUs).

**Methods:**

We model the decision making problem as a Markov Decision Process, and use a batch RL method, *Fitted Q Iterations with Gradient Boosting Decision Tree*, to learn a suitable ventilator weaning policy from real trajectories in retrospective ICU data. A Bayesian inverse RL method is then applied to infer the latent reward functions in terms of weights in trading off various aspects of evaluation criterion. We then evaluate how the policy learned using the Bayesian inverse RL method matches the policy given by clinicians, as compared to other policies learned with fixed reward functions.

**Results:**

Results show that the inverse RL method is capable of extracting meaningful indicators for recommending extubation readiness and sedative dosage, indicating that clinicians pay more attention to patients’ physiological stability (e.g., heart rate and respiration rate), rather than oxygenation criteria (*FiO*_2_, PEEP and *SpO*_2_) which is supported by previous RL methods. Moreover, by discovering the optimal weights, new effective treatment protocols can be suggested.

**Conclusions:**

Inverse RL is an effective approach to discovering clinicians’ underlying reward functions for designing better treatment protocols in the ventilation weaning and sedative dosing in future ICUs.

## Background

Emerging in recent years as a powerful trend and paradigm in machine learning, *reinforcement learning* (RL) [1] has achieved tremendous achievements in solving complex sequential decision making problems in various health care domains, including treatment in HIV [2], cancer [3], diabetics [4], anaemia [5], schizophrenia [6], epilepsy [7], anesthesia [8], and sepsis [9], just to name a few. However, all the existing RL applications are grounded on an available reward function, either in a numerical or an qualitative form, to indicate the goal of treatments by clinicians. Explicitly specifying such a reward function not only heavily requires prior domain knowledge, but also relies on clinicians’ personal experience that varies from one to another. Thus, consistent learning performance might not be always guaranteed. In fact, even some components of reward information can be manually defined, it is usually ambiguous to specify exactly how such components should be traded off in an explicit and effective manner. As such, in situations when no explicit information is available regarding the reward function, it is then necessary to consider an approach to RL whereby the reward function can be learned from a set of presumably optimal treatment trajectories using retrospective real medical data.

The problem of deriving a reward function from observed behaviors or data is referred to as *inverse reinforcement learning* (IRL) [10], which has received an increasingly high interest by researchers in the past few years. These methods have achieved substantial success in a wide range of applications, from imitation of autonomous driving behaviors [11, 12], control of robot navigation [13] to high dimensional motion analysis [14].

Despite the above tremendous progress, there is surprisingly quite limited work on applying IRL approaches in clinical settings. We conjecture that such an absence might be due to the inherent complexity of clinical data and its associated uncertainties in the decision making process. In fact, medical domains present special challenges with respect to data acquisition, analysis, interpretation and presentation of these data in a clinically relevant and usable format [15]. Medical data are usually noisy, biased and incomplete, posing significant challenges for existing RL methods. For example, many studies are conducted with patients who fail to complete part of the study, or, because of the finite duration of most studies, there is often no information about outcomes after some fixed period of time. The missing or censoring data will tend to increase the variance of estimates of the value function and the policy in RL [16]. This problem becomes even more severe in the case of IRL, where algorithms not only need to learn a policy using RL, but also need to learn a reward function using data characterized by the above notorious features. The errors brought in during the policy learning and reward learning intertwine with each other in IRL, potentially leading to divergent solutions that are of little use in practical clinical applications [17].

In this paper, we aim to apply IRL methods in solving a specific clinical decision making problem in ICUs, i.e., the management of invasive mechanical ventilation, and the regulation of sedation and analgesia during ventilation [18]. Since prolonged dependence on mechanical ventilation can cause higher hospital cost while increased risk of complications may occur if premature extubation is conducted, it is pressing to develop an effective protocol for weaning patients off from a ventilator by properly trading off these two aspects and making optimal sedative dosing during this process. By using sets of real treatment trajectories, we infer the reward functions that clinicians have in mind during their decisions of mechanical ventilation and sedative dosing in ICUs. Experiments verify the effectiveness of IRL in discovering clinicians’ underlying reward functions, which are then exploited for designing better new treatment protocols in ICUs.

## Related work

With the development in ubiquitous monitoring techniques, a plethora of ICU data has been generated in a variety of formats such as free-text clinical notes, images, physiological waveforms, and vital sign time series, enable optimal diagnose, treat and mortality prediction of a patient in ICUs [15]. Thus far, a great deal of theoretical or experimental studies have employed RL techniques and models for decision support in critical care. Nemati et al. developed deep RL algorithms that learn an optimal heparin dosing policy from real trails in large electronic medical records [19, 20]. Sandu et al. studied the blood pressure regulation problem in post cardiac surgery patients using RL [21]. Padmanabhan et al. resorted to RL for the control of continuous intravenous infusion of propofol for ICU patients by both considering anesthetic effect and regulating the mean arterial pressure to a desired range [8]. Raghu et al. proposed an approach to deduce treatment policies for septic patients by using continuous deep RL methods [22], and Weng et al. applied RL to learn personalized optimal glycemic treatments for severely ill septic patients [9]. The most related work is that by Prasad et al., who applied batch RL algorithms, fitted Q iteration with extremely randomized trees, to determine the best weaning time of invasive mechanical ventilation, and the associated personalized sedative dosage [18]. Results demonstrate that the learned policies show promise in recommending weaning protocols with improved outcomes, in terms of minimizing rates of reintubation and regulating physiological stability. However, all these studies are built upon a well predefined reward function that requires heavy domain knowledge and manual engineering.

Ng and Russell first introduced IRL to describe the problem of recovering a reward function of an MDP from demonstrations [10]. Numerous IRL methods have been proposed afterwards, including *Apprenticeship Learning* [11], *Maximum Entropy IRL* [23], *Bayesian IRL* [24], and nonlinear representations of the reward function using Gaussian processes [25]. Most of these methods need to solve an RL problem in each step of reward learning, requiring an accurate model of the system’s dynamics that is either given a priori or can be estimated well enough from demonstrations. However, such accurate models are rarely available in clinical settings. How to guarantee the performance of the RL solutions in an IRL process is an unsolved issue in IRL applications, especially in clinical settings where the only available information is the observations of a clinician’s treatment data that are subject to unavoidable noise, bias and censoring issues.

## Methods

In this setion, we investigate the possibility of applying IRL approaches in solving complex clinical decision making problems, that is, automated weaning of mechanical ventilation and optimal sedative dosage in ICUs. To this end, the critical care data set and its preprocessing are introduced first. The decision making framework and its associated RL components are then discussed. Finally, an IRL method is applied to refer the reward functions used by clinicians.

### Preprocessing of critical care data

We use the Medical Information Mart for Intensive Care (MIMIC III) database [26], which is a large, freely accessible database comprising identified health-related information from nearly forty thousand distinct adult patients and eight thousand neonates who stayed in critical care units of the Beth Israel Deaconess Medical Center between 2001 and 2012. The database is mainly for academic and industrial research purpose, offering a variety forms of data in critical care including demographics, vital signs, laboratory tests, diagnoses, medications, and more.

To acquire the data for our purpose, we first extract 8860 admissions from adult patients undergoing invasive ventilation in MIMIC III database. In order to focus on weaning ventilation and sedative dosing, we further filter these data by excluding those admissions when the patient was kept under ventilation for less than 24 hours, or unsuccessfully discharged from the hospital by the end of the admission. This allows us to exclude those admissions when ventilation was applied during a short period of time (e.g., after a surgery), or when a patient expired in the ICUs due to many other factors unrelated merely to the ventilator support and sedative dosing [18]. In this paper, we mainly focus on learning policies for weaning of ventilation and sedative dosing, thus, prolonged ventilation, administration of unsuccessful spontaneous breathing trials, or reintubation within the same admission are considered to be the main factors during decision makings.

**Fig. 1:**
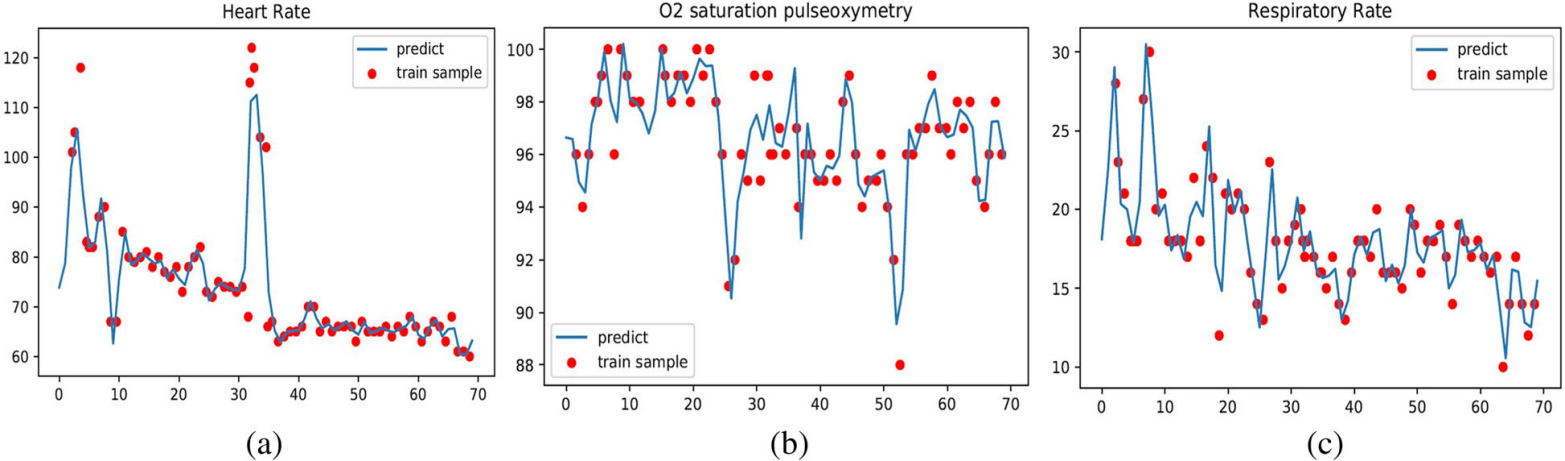
Example trajectories of three vital signs (Heart Rate, *SpO*_2_ and Respiratory Rate) after preprocessing. **a** Heart Rate **b** Sp0 _2_**c** Respiratory Rate

Data in critical care are characterized by issues of compartmentalization, corruption and complexity [15]. Measurements of vital signals and lab results may be irregular, sparse, and error-prone. Some physiological parameters are taken several times an hour, such as heart rate or respiratory rate, while other physiological parameters are measured only once in several hours, such as arterial pH or oxygen pressure. Changes in body state occur all the time, and naive methods for interpolation do not meet the necessary accuracy at higher temporal resolutions. Therefore, we use *support vector machines* (SVM) to fit the physiological parameters according to measurement time. After preprocessing, we obtain complete data for each patient, at a temporal resolution of 10 minutes, from admission time to discharge time. Figure 1 shows example trajectories of three vital signs (Heart Rate, *SpO*_2_ and Respiratory Rate) after preprocessing. It shows that the predicted trajectories can fit the sample trajectories at a high accuracy.

### MDP formulation and the RL solution

The decision making process of our problem is modeled as a *Markov Decision Process* (MDP) by a tuple of 〈*S,A,P,R*〉, in which *s*_*t*_∈*S* is a patient’s state at time *t*, *a*_*t*_∈*A* is the action made by clinicians at time *t*, *P*(*s*_*t*+1_|*s*_*t*_,*a*_*t*_) is the probability of the next state after given the current state and action, and *r*(*s*_*t*_,*a*_*t*_)∈*R* is the observed reward following a transition at time step *t*. The goal of an RL agent is to learn a policy to maximize the expected accumulated reward over time horizon *T* by:

$$R^{\pi}(s_{t})={\lim}_{T \rightarrow \infty} E_{s_{t+1}|s_{t},\pi(s_{t})} \sum\limits^{T}_{t+1} \gamma^{t} r(s_{t},a_{t}), $$

where the discount factor *γ* determines the relative weight of immediate and long-term rewards.

The patient’s response to sedation and extubation depends on many different factors, from demographic characteristics, pre-existing conditions, and comorbidities to specific time-varying vital signs, and there is considerable variability in clinical opinion on the extent of the influence of different factors [18]. We extracted the most important 13-dimensional features, including *respiration rate*, *heart rate*, *arterial pH*, *positive end-expiratory pressure* (PEEP) set, *oxygen saturation pulse oxymetry* (*SpO*_2_), *inspired oxygen fraction* (*FiO*_2_), *arterial oxygen partial pressure*, *plateau pressure*, *average airway pressure*, *mean non-invasive blood pressure*, *body weight* (kg), *age*, and *ventilation*. In designing the set of actions, two actions are defined to indicate a patient off or on the ventilator, respectively. As for the sedative, we focus on a commonly used sedative, the *propofol*, and separate the dosage into four different levels of sedation. Thus, the action set defined includes eight combinational actions in total.

The formulation of reward function is the key in successful applications of RL approaches. Here, we design a reward function *r*_*t*+1_, under the guidance of clinicians at the Hospital of University of Pennsylvania (HUP). Current extubation guidelines at HUP must meet the following two main conditions: the *physiological stability* (respiratory rate ≤ 30, heart rate ≤ 130, and arterial pH ≥ 7.3), and the *oxygenation criteria* (PEEP (cm *H*_2_*O*) ≤8), *SpO*_2_ (%) ≥ 88, and *FiO*_2_ (%) ≤50). Following previous work [18], the reward function *r*_*t*+1_ is defined as $r_{t+1} = r^{vitals}_{t+1} + r^{{vent off}}_{t+1} + r^{{vent on}}_{t+1}$ to reward physiological stability and successful extubation while penalizing adverse events (i.e., failed *spontaneous breathing trials* SBTs or reintubation).

Reward component $r^{{vitals}}_{t+1}$ evaluates how the actions perform regarding the patient’s physiological stability in terms of staying within a reasonable range and having not changed drastically:

$$ \begin{aligned} r^{{vitals}}_{t+1} \!=& C_{1} \sum\limits_{v_{t}^{sta}} \left[\!\frac{1}{1 + e^{-\left(v_{t}^{sta} - v_{{min}}^{sta}\right)}} - \frac{1}{1 + e^{-\left(v_{t}^{sta} - v_{{max}}^{sta}\right)}} + \frac{1}{2} \right] \\ \left. \right. &- C_{2} \left[ \max\left(0, \frac{|v_{t+1}^{sta} - v_{t}^{sta}|}{v_{t}^{sta}}- 0.2\right) \right], \end{aligned} $$

where values $v_{t}^{sta}$ are the measurements of physiological vitals in the state definition (i.e., respiratory rate, heart Rate, and arterial pH) at time *t*, which are believed to be indicative of physiological stability. When $v_{t}^{sta} \in \left [v_{min}^{sta},v_{max}^{sta}\right ]$, the patient is considered to be in a physiologically stable state. The second part on the right indicates the negative feedback when consecutive measurements had a sharp change, which is detrimental to the patient.

Reward component $r^{{vent off}}_{t+1}$ evaluates the performance of weaning ventilation at time *t*+1:

$$\begin{array}{*{20}l} & r^{{vent\ off}}_{t+1} = \mathbbm{1}_{[s_{t+1}(\text{vent on}) = 0]} \left[ C_{3} \cdot \mathbbm{1}_{[s_{t}(\text{vent on}) = 1]} \right. \\ &\qquad \quad \left. + C_{4}\cdot \mathbbm{1}_{[s_{t}(\text{vent on}) = 0]} - C_{5}\sum\limits_{v_{t}^{{ext}}}\mathbbm{1}_{\left[v^{{ext}}_{t} \,>\, v^{{ext}}_{{max}} \,||\, v^{{ext}}_{t} \,<\, v^{{ext}}_{{min}}\right]} \right]\!, \end{array} $$

where $v_{t}^{ext}$ are parameters related to the conditions for extubation (i.e., *FiO*_2_, *SpO*_2_, PEEP), and $\mathbbm {1}_{[con.]}$ is an indicator function that returns 1 if the condition *con*. is true, and 0 otherwise. If $v_{t}^{ext} \notin \left [v_{nim}^{ext},v_{max}^{ext}\right ]$, which means the condition is not suitable for extubation, the agent will get negative rewards when extubation has been conducted. Otherwise, a positive reward will be given at the time of successful extubation (i.e., the *C*_3_ component).

The last reward component $r^{{vent on}}_{t+1}$ simply indicates the cost for each additional hour spent on the ventilator:

$$\begin{array}{*{20}l} r^{{vent\ on}}_{t+1} =& \mathbbm{1}_{[s_{t+1}(\text{vent on}) = 1]} \left[C_{6}\cdot\mathbbm{1}_{[s_{t}(\text{vent on}) = 1]}\right. \\ &\left. - C_{7} \cdot\mathbbm{1}_{[s_{t}(\text{vent on}) = 0]}\right]. \end{array} $$

Constants *C*_1_ to *C*_7_ are weights of the reward function (*C*_1_+…+…*C*_7_=1), which determine the relative importance of each reward component. Given a predefined weight vector, existing RL methods can be applied to learn an optimal policy for the decision making problem. Due to its data efficiency, we adopt an off-policy batch-mode RL method, the *Fitted Q-iteration* (FQI) [27], to solve the learning problem. FQI uses a set of one-step transition tuples $\mathcal {F} = \left \{\left (\left \langle s_{t}^{n}, a_{t}^{n}, s_{t+1}^{n}\right \rangle, r^{n}_{t+1}\right), n=1,\ldots,|\mathcal {F}|\right \}$ to learn a sequence of function approximators of the Q values (i.e., the expected value of state-action pairs) by iteratively solving supervised learning problems. The *Q* values are updated at each iteration according to the Bellman equation: $\hat {Q}_{k}(s_{t}, a_{t}) \leftarrow r_{t+1} + \gamma {\underset {a \in \mathcal {A}}{\max }}\, \hat {Q}_{k-1}(s_{t+1}, a)$, where $\hat {Q}_{1}(s_{t}, a_{t}) = r_{t+1}$. The approximation of the optimal policy after *K* iterations is then given by:

$$\hat{\pi}^{*}(s) = {\underset{a \in \mathcal{A}}{\arg\max}}\,\hat{Q}_{K}(s,a).$$

Although various existing supervised learning methods are available for regression in FQI, including kernel-based methods and decision trees, in this paper, we use the *Gradient Boosting Decision Tree* (GBDT) [28] method as the regression method due to its supreme performance in generalization.

### A Bayesian IRL solution

The direct application of RL approaches requires predefined weight parameters such that a feasible policy can be learned. Although, generally, the reward function can be formulated according to some domain knowledge, in many situations, an explicit formulation of reward functions is difficult or even impossible, even with the guidance of experts. In response to this problem, an apprenticeship learning algorithm was proposed [11], which learns the reward function from the trajectory of an expert’s demonstration, so that the learned policy can match the expert’s policy most [11]. Although the basic reward components for the ventilation weaning and sedative dosing problem in ICUs have been formulated based on the guidance of expert doctors, how to derive the weights to trade off these components is still a challenging issue to be resolved.


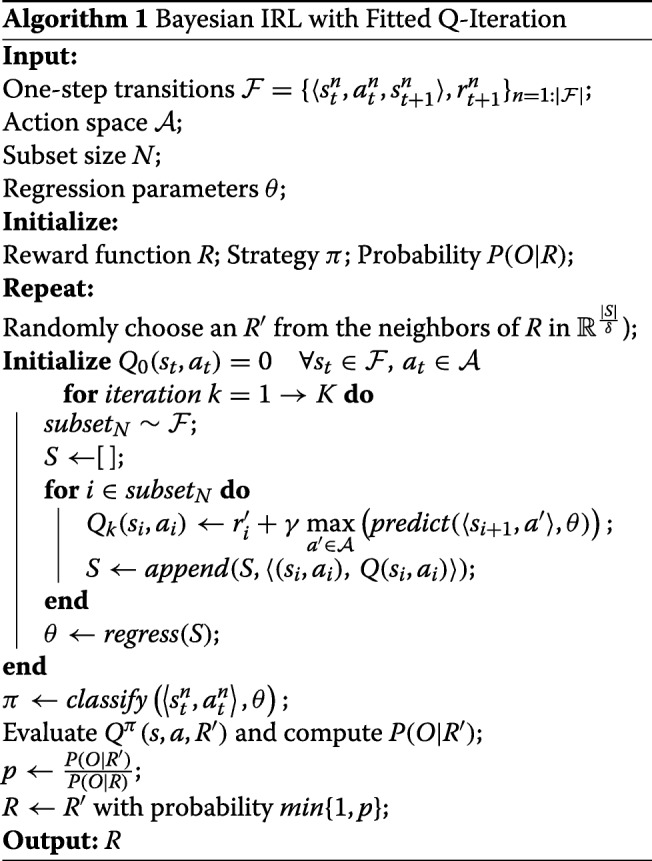


To this end, we first intend to use the apprenticeship learning algorithm to learn the complete reward function (i.e., values for *C*_1_,…,*C*_7_) from the cases treated by expert clinicians and then optimize the policies learned by using this reward function. To use apprenticeship learning algorithm, a concept called *feature expectation* should be defined, which can be simply understood as the expectation of the corresponding environmental feature under the current policy. The algorithm then proceeds as follows. The reward function is initialized first, randomly or preferentially according to some prior knowledge, and then any RL algorithm can be used to compute an intermediate policy. By assuming an accurate model of the system’s dynamics that can be either given a priori or can be estimated well enough from the data trajectories, the feature expectation for the intermediate policy can be calculated. After that, it calculates the weight of the reward function to ensure that the closest feature expectation between the expert policy and the intermediate policy be maximized. Then the new reward function can be applied to compute a new policy and a new feature expectation. This process iterates until the resulting policy is close enough to the expert’s policy.

However, after applying apprenticeship learning algorithm in the ICU problem, the learned policy could not converge at all. After a deeper analysis, we found that the reason was attributed to correlation of state features in the reward function with the length of patient’s stay in ICUs and the number of inbutation and exbutation. These factors are affected by many other issues such as the patient’s personal situation, the degree of shortage in ICU wards, and the personal treatment strategy preference, which cannot effectively distinguish the expert’s polices and non-expert policies. Particularly, naive apprenticeship learning algorithms that are built on the comparison of feature expectations are unsuited for problems of bivariate features with a varying length of trajectories, since this would cause significant bias in computing the expectations for such features, leading to divergence of final learning performance.

**Fig. 2:**
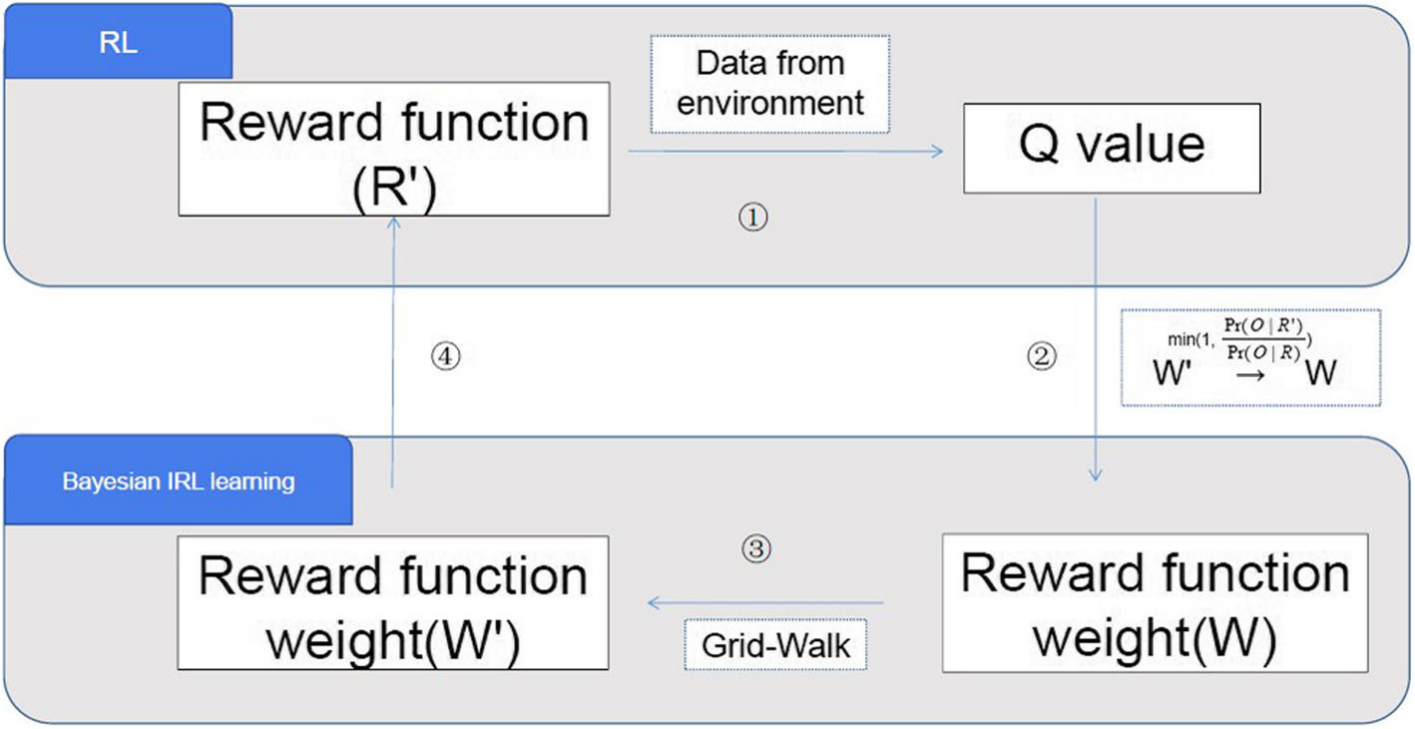
The process of IBL

To avoid the above problems, we exploited the Bayesian IRL algorithm [24] to learn the reward function. The whole learning procedure is given by Fig. 2. We assume that under the reward value function R, the possibility of the agent performing the expert trajectory *O*={(*s*_1_,*a*_1_),…(*s*_*k*_,*a*_*k*_)} is given by $Pr(O|R) = \prod ^{k}_{i = 1}Pr((s_{i},a_{i})|R)$, in which the possibility for each (*s*_*i*_,*a*_*i*_) is assumed to follow the Boltzmann distribution as $Pr((s_{i},a_{i})|R) = \frac {1}{C_{i}}e^{\alpha Q^{*}(s_{i},a_{i},R)}$, where *Q*^∗^(*s,a,R*) is a potential function (the action value function) under the optimal policy for *R*, $C_{i}={\sum }_{a \in A}e^{\alpha Q^{*}(s_{i},a,R)}$ is the normalization constant, and *α* is a parameter to adjust the possibility of the expert’s choice of action. Combined with the prior distribution of the reward function R, the posterior probability of R under the observation and action sequence *O* can be computed using Bayes’ theorem as $ Pr(R|O)=\frac {1}{Z}e^{\alpha {\sum }_{i}Q^{*}(s_{i},a_{i},R)}Pr(R)$, where Z is another normalization constant. When no other information is given, we assume that reward value function *Pr*(*R*) obeys a uniform distribution.

In order to compute the posterior distribution of *R*, we use a Markov Chain Monte Carlo (MCMC) algorithm (GridWalk) as the sampling method, which generates a Markov chain on the intersection points of a grid of length *δ* in the region $\mathbb {R}^{|S|}$ (denoted as $\mathbb {R}^{\frac {|S|}{\delta }}$). Algorithm 1 gives the main procedure of the Bayesian IRL method with FQI as the RL algorithm in each inner iteration of policy learning.

## Results

As there are six commonly used sedatives in the MIMIC III data set, we extract 707 admissions that the *propofol* was applied as the sedative. These data are then split into the training set with 566 admissions and test set with the remaining 141 admissions. The radial basis function is used as the kernel function in SVM with regularization coefficient C being 25. After data preprocessing, 285.5 and 150.1 thousands one-step transitions are generated in the training set and test set, respectively. In order to ensure faster training speed, we take 10000 one-step transitions for training in each iteration of FQI. The number of boosting stages is 100, and learning rate is 0.1. All the samples are used for fitting the individual base learners, and the least squares loss function is to be optimized. For each base learner, all the features are considered when looking for the best split. The maximum depth is 3, the minimum number of samples required to split an internal node is 2, and at least one sample is required to be at a leaf node. Other hyper-parameters are set as default values.

**Fig. 3:**
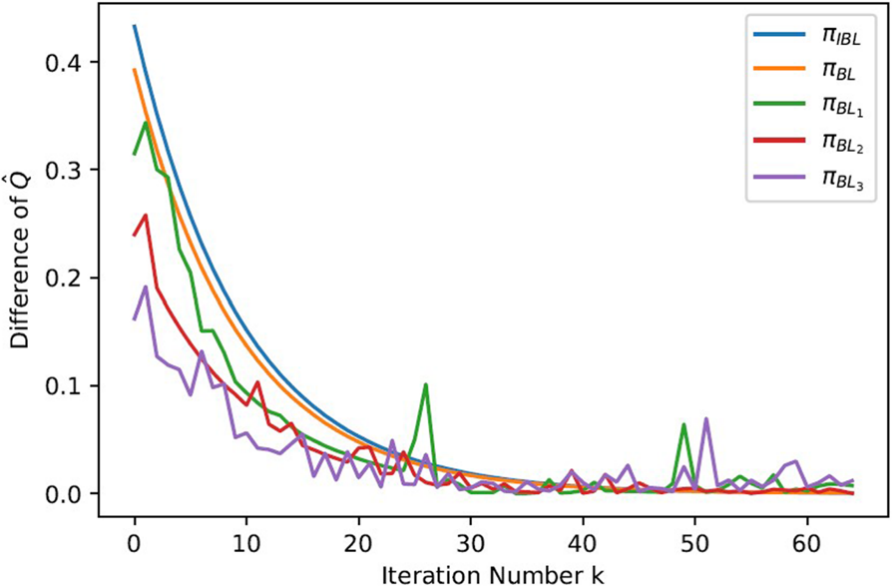
Convergence of $\hat {Q}$ using FQI-GBDT and the inverse FQI-GBDT methods

First, we would like to evaluate whether the FQI method combined with GBDT as the regressor, and its inverse version are capable of learning any effective solutions. For each weight *C*_*i*_(*i*∈{1,…,7}), we constrain its value in between [0,1] to indicate different levels of importance. We test FQI-GBDT using a weight vector of [1/7,1/7,1/7,1/7,1/7,1/7,1/7] (i.e., *π*_*BL*_), and the other three different weight vectors that are generated randomly from the range of [0,1], corresponding to $\phantom {\dot {i}\!}\pi _{{BL}_{1}}$, $\phantom {\dot {i}\!}\pi _{{BL}_{2}}$, and $\phantom {\dot {i}\!}\pi _{{BL}_{3}}$, respectively. Table 1 presents the parameter settings for RL policies. In order to use the Bayesian IRL with FQI-GBDT, we choose the initial weight vector as [0,0,0,0,0,0,0] to indicate none prior knowledge about the value functions. After each exploration of the weights in the IRL process, the weights are then normalized such that their sum is equal to 1. Figure 3 plots the convergence of the learning processes in terms of difference of Q values in two consecutive iterations. Both the RL methods and the IRL method are capable of achieving a convergence after around 40 iterations, which verifies the effectiveness of the application of RL and IRL methods in solving the ventilation and sedative dosing problems in ICUs. Since IRL method involves a process of estimating the reward function during learning, it can bring about a more efficient and robust learning process than the RL methods that are based on predefined fixed reward functions.

**Table 1:** Weight vectors for different RL policies

Policy	Weight of reward function
*π*_*BL*_	[1/7,1/7,1/7,1/7,1/7,1/7,1/7]
$\phantom {\dot {i}\!}\pi _{{BL}_{1}}$	[0.14,0.24,0.15,0.19,0.07,0.07,0.14]
$\phantom {\dot {i}\!}\pi _{{BL}_{2}}$	[0.08,0.17,0.16,0.18,0.29,0.10,0.02]
$\phantom {\dot {i}\!}\pi _{{BL}_{3}}$	[0.07,0.19,0.12,0.21,0.26,0.04,0.11]

**Fig. 4:**
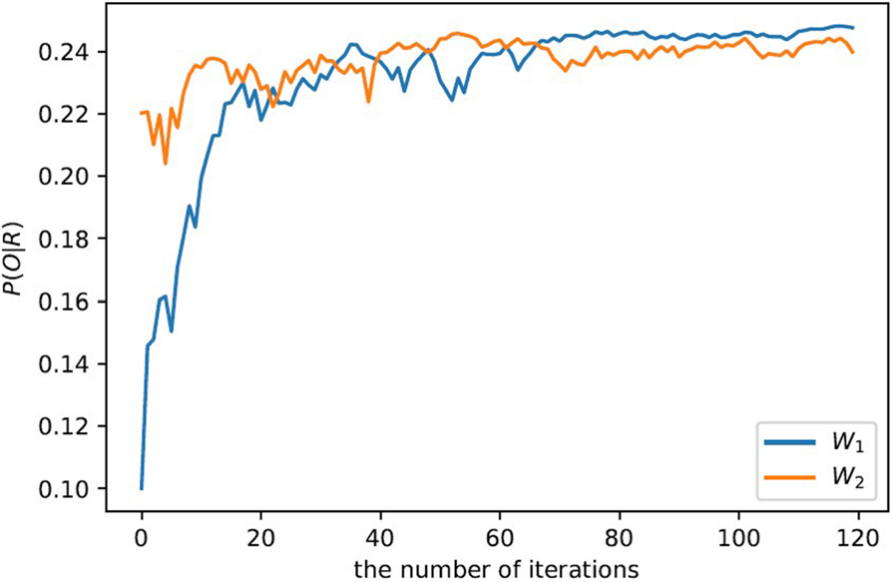
The convergence of P(*O*|*R*) using different initial values of the weights

Figure 4 plots the convergence of probability P(*O*|*R*) using Bayesian IRL, where *W*_1_=[0,0,0,0,0,0,0] and *W*_2_:[1/7,1/7,1/7,1/7,1/7,1/7,1/7]. As the number of iterations increases, the policies learned by using *π*_*IBL*_ are getting closer to the expert’s policy. However, weight *W*_2_ enables a better initial performance than *W*_1_ due to less exploration in the reward function space. Note that P(*O*|*R*) is not a probability converging to 1, since it is a proportion value that an action’s potential function (i.e., Q function) accounts for the potential functions of all the actions. Results in Fig. 4 thus indicate that the efficiency of a Bayesain IRL method closely depends on the initial weights of the reward functions. If some prior knowledge about the reward functions is available, learning efficiency can be greatly improved by initializing weights to those specified by this prior knowledge. Enabling the integration of domain knowledge into the learning process for performance improvement is also a major merit of Bayesain IRL methods.

In order to assess how well the policies learned match the true policies of the doctors, we validate all the policies on the test set of real medical data. As shown in Table 2, the performance of RL methods heavily depends on the choice of initial reward weights. Policy *π*_*BL*_ matches 53.5% of the joint action of doctors, with 99.6% consistency in ventilation action and 53.9% in sedative action, while policy $\phantom {\dot {i}\!}\pi _{{BL}_{2}}$ can only matches averagely 14.1% of the joint action. The IRL method is consistent with doctors’ actions in ventilation by 99.7% and in sedative dosing by 54.2%, achieving an overall consistency of 53.9%.

**Table 2:** The correctness of learned polices using RL and IRL methods in the test data set

Policy	Overall Action	Ventilation	Sedative
*π*_*IBL*_	53.9*%*	99.7*%*	54.2*%*
*π*_*BL*_	53.5*%*	99.6*%*	53.9*%*
$\phantom {\dot {i}\!}\pi _{{BL}_{1}}$	23.5*%*	45.7*%*	51.0*%*
$\phantom {\dot {i}\!}\pi _{{BL}_{2}}$	14.1*%*	35.5*%*	39.1*%*
$\phantom {\dot {i}\!}\pi _{{BL}_{3}}$	17.2*%*	34.9*%*	54.1*%*

We further divide the test data set into two main sub-groups: expert data set, in which inbutation was conducted only once and the SBTs were successful, and non-expert data set in which inbutation was conducted only once but the SBTs failed (i.e., Ordinary Single Intubation Data) or inbutation was conducted more than once (i.e., Multiple Intubation Data). The latter two data sets are considered to be non-expert data sets because wrong decisions of weaning the ventilation or sedative dosing caused the failure of SBTs or inbutation more than once. Table 3 shows the results of *π*_*IBL*_ and *π*_*BL*_ in these test data sets in terms of match of sedative dosing actions. As *π*_*BL*_ is the best policy among all the RL polices, it can achieve a comparable correctness against *π*_*IBL*_. However, the correctness of the non-expert sets, particularly the multiple intubation data set, is much higher than the expert test set. This is because it is more difficult to derive the experts’ reward functions compared with non-experts, since non-experts’ reward functions (i.e., clinical decisions) usually deviate far away from the true ones expressed by experts. The larger bias thus enables IRL methods to explore the whole reward function space more easily.

**Table 3:** The correctness of sedative dosing polices using RL and IRL methods in the test data set

Policy	Expert Data	Ordinary Single Intubation Data	Multiple Intubation Data
*π*_*IBL*_	44.5*%*	48.5*%*	63.4*%*
*π*_*BL*_	44.4*%*	48.4*%*	62.8*%*

## Discussion

**Fig. 5:**
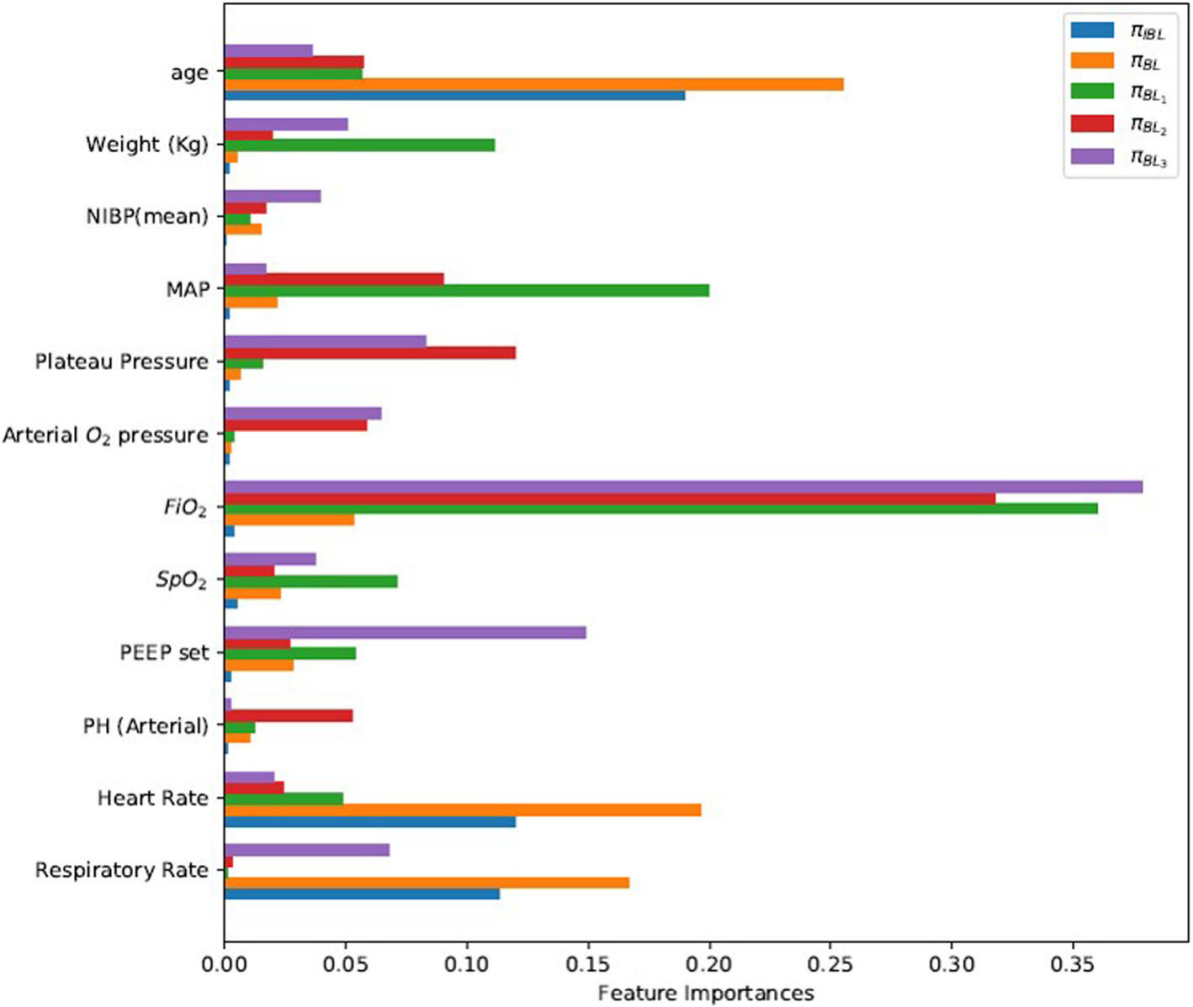
Comparison of feature importance using different RL and IRL policies

Current extubation guidelines provide precise conditions for clinicians to determine when extubation is most preferable. However, the priorities of these conditions are usually based on clinicians’ personal experience, thus having not been explicitly specified. Figure 5 compares the importance of patients’ physiological indicators and ventilator parameters using the policies learned by the four RL methods and IRL method. It is clear that the feature importance of the policies learned by different reward weights and learning methods differ from each other a lot. For example, the top three important features are (*FiO*_2_, MAP and age), (*FiO*_2_, MAP and PEEP set), and (*FiO*_2_, MAP and *SpO*_2_) for policy $\phantom {\dot {i}\!}\pi _{{BL}_{3}}$, $\phantom {\dot {i}\!}\pi _{{BL}_{2}}$, and $\phantom {\dot {i}\!}\pi _{{BL}_{1}}$, respectively. However, results in Fig. 3 show that the three RL methods perform poorly in terms of slow convergence rate and unstable learning process, indicating the limitations of such feature priorities.

**Fig. 6:**
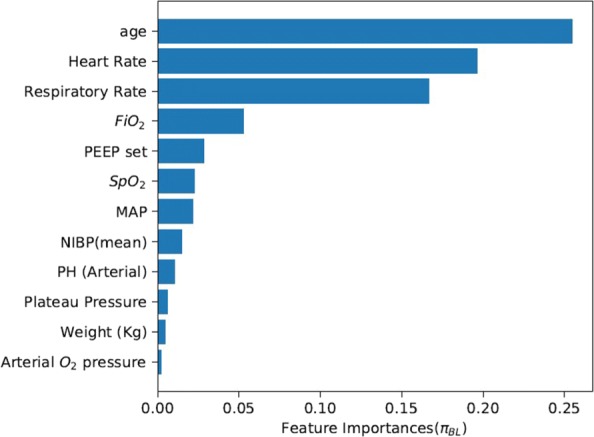
Feature importance using *π*_*BL*_

**Fig. 7:**
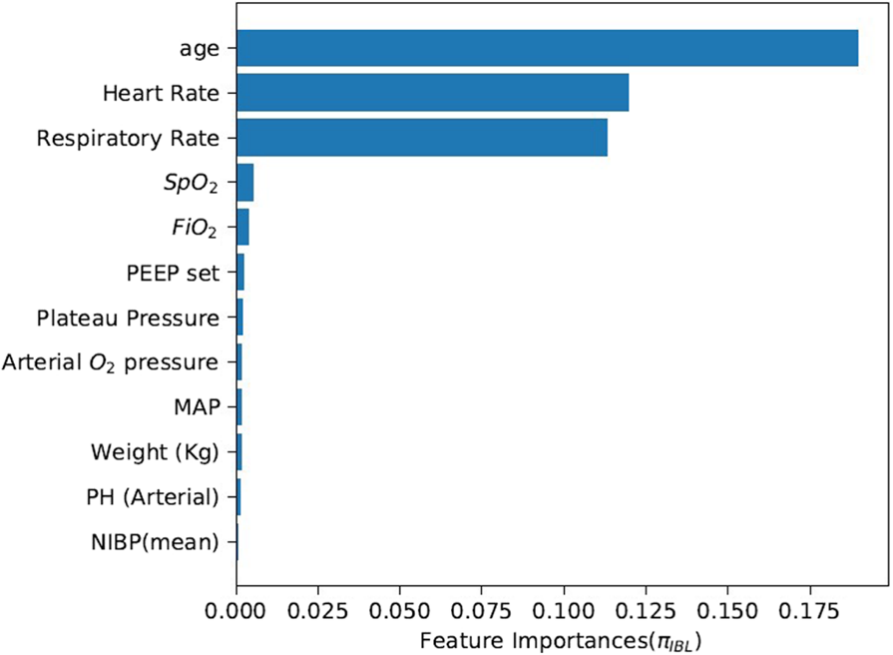
Feature importance using *π*_*IBL*_

To provide a deeper insight, we compared the importance of related features using the two more efficient methods of *π*_*BL*_ and *π*_*IBL*_. Figures 6 and 7 show that the importance of related features shares quite similar patterns. The top three important features are age, heart rate and respiratory rate, and these three features together account for a large proportion of all the features. Particularly, the age of a patient is strongly correlated with the patient’s ability to recover, and thus is given the highest priority when considering ventilation and sedative treatment policies in ICUs. Besides, heart rate and respiration rate are two main factors in maintaining physiological stability. Paying special attention to these factors is contradictory to the other three RL methods (i.e., $\phantom {\dot {i}\!}\pi _{{BL}_{1}}$, $\phantom {\dot {i}\!}\pi _{{BL}_{2}}$, and $\phantom {\dot {i}\!}\pi _{{BL}_{3}}$) that pay more attention to oxygenation criteria of *FiO*_2_, PEEP and *SpO*_2_.

Although *π*_*IBL*_ and *π*_*BL*_ methods share similar learning performance, surprisingly, the learned weights differ a lot. The weights of the reward function using *π*_*IBL*_ is finally stabilized at [0.26,0.06,0.18,0.12,0.08,0.28,0.02]. Compared with the weights [1/7,1/7,1/7,1/7,1/7,1/7,1/7] using the *π*_*BL*_ method, weights *C*_2_, *C*_5_ and *C*_7_ using *π*_*IBL*_ are much smaller, while weights *C*_1_ and *C*_6_ are much larger. This indicates that, rather than considering all the seven factors equally, doctors give higher priorities to the patient’s physiological stability in terms of staying within a reasonable range (i.e., higher *C*_1_), and the cost for each additional hour spent on the ventilator (i.e., higher *C*_6_), but lower priorities to other factors. These results suggest helpful insights into the development of new effective treatment protocols for intelligent ventilation and sedative dosing in ICUs.

## Conclusions

In this work, a data-driven approach is proposed to the optimization of weaning mechanical ventilation and sedative dosing for patients in ICUs. We model the decision making problem as an MDP, and use a batch RL method, FQI with GBDT, to learn a suitable ventilator weaning policy from real trajectories in historical ICU data. A Bayesian IRL method is then applied to infer the latent reward functions in terms of weights in trading off various aspects of evaluation criterion. We demonstrate that the approach is capable of extracting meaningful indicators for recommending extubation readiness and sedative dosage, on average outperforming direct RL methods in terms of regulation of vitals and reintubations. Moreover, by discovering the optimal weights using IRL methods, new effective treatment protocols can be suggested in the intelligent decision making of ventilation weaning and sedative dosing in future ICUs.

Although our work has verified the effectiveness of IRL methods in complex clinical decision making problems, there are a number of issues that need to be carefully resolved before these methods can be meaningfully implemented in a clinical setting. First, in this paper, the two main processes of data preprocessing and data learning are conducted separately. There is no doubt that the errors brought in the preprocessing process will affect the learning accuracy in the data learning period. It is thus necessary to enable IRL methods to directly work on the raw noisy and incomplete data. Moreover, most existing IRL methods require an accurate model to be given beforehand or estimated from data. This is infeasible when such a model is lacking or accurate estimation of the model is infeasible directly from expert demonstrations, particularly in clinical settings where the model always involves a large volume of continuous states and actions. It is thus valuable to apply IRL methods that are capable of estimating the rewards and model dynamics simultaneously. Some theoretical research on IRL [17, 29] has investigated these issues recently and can be investigated in the clinical settings here. These issues are left for our future work.
